# G Protein-Coupled Receptors and Ion Channels Involvement in Cisplatin-Induced Peripheral Neuropathy: A Review of Preclinical Studies

**DOI:** 10.3390/cancers16030580

**Published:** 2024-01-30

**Authors:** Gabriela Becker, Samuel Felipe Atuati, Sara Marchesan Oliveira

**Affiliations:** Laboratory of Neurotoxicity and Psychopharmacology—Pain Research Group, Graduate Program in Biological Sciences: Toxicological Biochemistry, Centre of Natural and Exact Sciences, Federal University of Santa Maria, Camobi, Santa Maria 97105-900, RS, Brazil; gabriela.becker@acad.ufsm.br (G.B.); samuel.atuati@acad.ufsm.br (S.F.A.)

**Keywords:** chemotherapy, cancer, neuropathic pain, kinins, cannabinoids, voltage-gated channels, transient receptor potential

## Abstract

**Simple Summary:**

Cisplatin is a chemotherapy drug extensively used to treat cancer, improving the survival of cancer patients. Although effective for this purpose, its use is accompanied by notorious severe adverse effects that significantly reduce patient’s quality of life. This review will discuss the main impact of chemotherapy on the peripheral nervous system, a clinical condition known as chemotherapy-induced peripheral neuropathy, with emphasis on cisplatin. Furthermore, the review encompasses an analysis of the role played by G protein-coupled receptors and ion channels in cisplatin-induced peripheral neuropathy, extracting insights from preclinical experimental studies.

**Abstract:**

Cisplatin is a platinum-based chemotherapy drug widely used to treat various solid tumours. Although it is effective in anti-cancer therapy, many patients develop peripheral neuropathy during and after cisplatin treatment. Peripheral neuropathy results from lesions or diseases in the peripheral somatosensory nervous system and is a significant cause of debilitation and suffering in patients. In recent years, preclinical studies have been conducted to elucidate the mechanisms involved in chemotherapy-induced peripheral neuropathic pain, as well as to promote new therapeutic targets since current treatments are ineffective and are associated with adverse effects. G-protein coupled receptors and ion channels play a significant role in pain processing and may represent promising targets for improving the management of cisplatin-induced neuropathic pain. This review describes the role of G protein-coupled receptors and ion channels in cisplatin-induced pain, analysing preclinical experimental studies that investigated the role of each receptor subtype in the modulation of cisplatin-induced pain.

## 1. Introduction

Neuropathic pain is a maladaptive condition resulting from a lesion or disease in the somatosensory nervous system [1,2]. Despite the estimate that neuropathic pain may affect 7 to 10% of the world’s population, data can be misleading due to the difficulty in defining a precise diagnosis and a broad epidemiological survey. The trend is a considerable increase due to higher rates of population ageing, diabetes, and cancer [3,4]. Neuropathic pain may be manifested as acute pain and be partially reversible, although the chronic pain state is more common [5]. Patients manifest ongoing and spontaneous pain-related symptoms, beginning as paraesthesia and dysesthesia, progressing to burning, numbness, pricking and tingling sensations, and electric shock-like pain, which may be accompanied by evoked pain such as sensitivity to mechanical and thermal stimulus [1,4,5].

Neuropathic pain can result from damage to the central or peripheral nervous system. Central neuropathic pain results from lesions in the spinal cord, stroke, and multiple sclerosis. Peripheral neuropathic pain may be caused by amputation (e.g., phantom limb pain), direct nerve damage, nerve compression, herpes zoster, human immunodeficiency virus (HIV), antiretrovirals, cancer, and chemotherapy agents [2,5]. However, some types of aetiology are challenging to define because they are related to mutual causes, such as neuropathic pain provoked by cancer and chemotherapy that simultaneously affect patients [6].

Cancer patients undergoing oncological treatment often experience pain resulting from the chemotherapy or the tumour itself. Neuropathic pain is one of the mechanisms that characterise chronic cancer pain, affecting 20% of cancer patients [7]. Similarly, chemotherapy-induced peripheral neuropathy (CIPN), a clinical condition of chronic peripheral neuropathic pain, is a prevalent phenomenon attributed to antineoplastic drugs, including platinum analogues and taxanes, affecting close to 90% of patients [8,9]. Denoted by its debilitating aspects, chronic pain negatively impacts individuals’ quality of life, inducing, besides painful symptoms, sleep and mood disturbances [6,9,10].

Due to the complexity of neuropathic pain in cancer patients, the clinical management of pain treatments is complex [6]. Another challenge in this context is that treatments for this purpose have low efficacy, as they provide only symptomatic resolution and cause multiple adverse effects [3,11]. Thus, understanding and elucidating the neurotoxic mechanisms involved in neuropathic pain may contribute to the study and application of new therapeutic interventions.

## 2. Cancer

Cancer is a highly debilitating condition and one of the major causes of death worldwide [12]. Remarkably, cancer represents a significant public health problem [12,13]. In 2020, around 19.3 million new cases and approximately 10 million deaths from cancer were recorded worldwide [12]. Among the various types, lung and breast cancers are the most prevalent and deadly [14,15,16]. Overall, cancer incidence continues to grow, and projections indicate a 47% increase in cases by the 2040s compared to 2020, representing around 28 million individuals affected [12]. The growth and ageing of the population are factors that reflect the increase in cancer cases [14,17].

Despite the high number of new cancer cases, patient survival has increased dramatically in recent years. This reflects the improvements in detection methods, allowing early diagnosis of many cases and, fundamentally, more effective and targeted oncologic treatments [17,18,19]. Nevertheless, the favourable statistics related to cancer survival are accompanied by physical and psychosocial adverse effects due to the cancer itself or its treatments [20]. Notably, pain is one of these events that affect cancer patients, which, along with other factors, contributes to reducing the quality of life and life expectancy of cancer patients and survivors [6,7,21].

The pain associated with cancer, in many patients, is the first sign of cancer and occurs at any time during the disease. Recent estimates indicate that an overall rate of 44.5% of cancer patients experience pain throughout all phases of the disease, while the prevalence is close to 55% at advanced stages [22]. Specifically, 40% of oncologic patients undergoing treatment report experiencing pain during and up to three months after treatment [23].

Cancer-related pain can result from the tumour itself (primary cancer or metastases), as a result of diagnostic (biopsies and resection) or therapeutic procedures (surgery, chemotherapy, and radiotherapy), and due to a combination of these factors [6,19]. Nevertheless, the emergence of neurotoxicity, manifested mainly as CIPN, represents a significant adverse effect associated with the extensive use of chemotherapy drugs to treat the most prevalent tumours [19,24].

## 3. Chemotherapy-Induced Peripheral Neuropathy (CIPN)

CIPN is a dose-limiting adverse effect of several commonly used chemotherapy drugs. The primary pharmacological classes related to the development of CIPN include the platinum-based antineoplastics drugs (mainly oxaliplatin and cisplatin), taxanes (paclitaxel, docetaxel), vinca alkaloids (vincristine and vinblastine), the proteasome inhibitors (bortezomib), and immunomodulatory drugs (thalidomide) [10,25,26].

Cisplatin, the pioneering platinum-derived compound, was approved by the Food and Drug Administration in 1978 for treating various solid tumours, employed independently or combined with other chemotherapeutic agents or radiotherapy [27,28]. Despite numerous advancements, cisplatin continues to be one of the most widely prescribed chemotherapy drugs, used in the treatment of a wide range of paediatric and adult malignancies such as ovarian, testis, lung, bladder, head and neck, advanced cervical cancer, lymphomas, and metastatic osteosarcomas, among others [27,29,30]. The antitumour action of cisplatin initially requires its intracellular bioactivation, where a highly reactive molecule binds to DNA via intra- and interstrand crosslinks and forms DNA-platinum adducts. The DNA-platinum adducts interfere with the proliferation of tumour cells by restricting DNA replication and transcription mechanisms, interrupting DNA synthesis and acting on signal transduction pathways, which results in programmed cell death [28,31,32].

Despite its effectiveness against various solid tumours, the non-specific nature of cisplatin and most chemotherapy drugs compromises the integrity of normal cells, resulting in the undesirable adverse effects commonly observed in cancer treatment [33]. In summary, chemotherapy drugs act in rapidly dividing cells and may affect healthy cells presenting this division profile, such as bone marrow cells or gastrointestinal mucosa. Intriguingly, this characteristic contrasts with neurons that are substantially affected by chemotherapy drugs, like cisplatin, leading to neurotoxicity and deleterious effects on the nervous system [20,34]. Chemotherapy-induced neurotoxicity occurs at the level of the central nervous system (known as “chemobrain”), enteric nervous system (referred to as enteric neuropathy), and peripheral nervous system (referred to as peripheral neuropathy) [34,35]. Herein, we will focus on the effects on the peripheral nervous system.

Peripheral sensory neurons are more susceptible to the toxic action of chemotherapy drugs, as in contrast to the central nervous system, the peripheral nervous system lacks a protective structure like the blood–brain barrier, allowing the diffusion of systemically administered drugs to the peripheral neurons [27,33,34,35]. Chemotherapy drugs affect the peripheral nervous system in different structures, including the axon, where the damage or loss results in axonopathy, or dorsal root ganglia (DRG) neurons, leading to neuronopathy/ganglionopathy [35,36].

DRG sensory neurons are the primary target of peripheral neurotoxicity resulting from platinum-based chemotherapy [31,36]. DRGs are vascularized by fenestrated capillaries, making them more permeable to circulating compounds and the main site of cisplatin accumulation [36]. In addition to the absence of the blood–brain barrier, two other reasons contribute to the increase in the amount of cisplatin in the DRG: increased absorption of platinum-compounds in the DRG and a decrease in the ability to metabolize cisplatin within the cells.

The increase in cisplatin absorption in the DRG occurs due to the expression of specific membrane transporters, called organic cation transporters (OCTs), which increase the transport of platinum compounds to cells. The second hypothesis suggests a decline in glutathione levels in DRG. Glutathione is a protein capable of binding and deactivating cisplatin, forming a complex that is subsequently eliminated from the cell [27,36,37]. 

**Figure 1:**
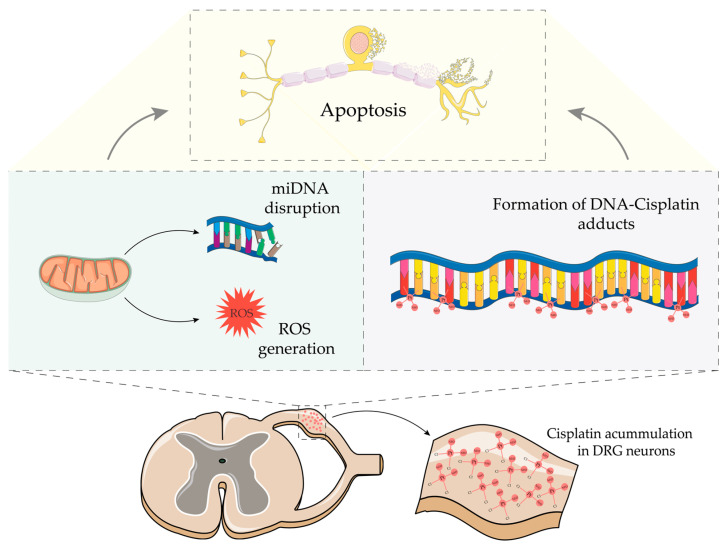
Schematic representation of the primary molecular mechanisms implicated in cisplatin-induced neurotoxicity. Once inside the neuron, cisplatin can bind to both nuclear and mitochondrial DNA. At the mitochondrial level, the DNA-cisplatin adducts disrupt their overall function, leading to increased ROS formation. Collectively, these events result in neuronal death via apoptosis. Parts of the Figure were drawn using pictures from Servier Medical Art by Servier, licensed under a Creative Commons Attribution 3.0 unported license.

In DRG, neurotoxicity cisplatin is associated with mitochondrial and nuclear DNA damage via forming nuclear and mitochondrial DNA-platin complexes (Figure 1). This process involves mitochondrial dysfunction, causing disturbances in protein synthesis and mitochondrial respiratory chain reactions, which lead to the overproduction of reactive oxygen species (ROS) and induce cellular oxidative stress, contributing as a component of neuronal apoptosis [27,31,38,39] (Figure 1). Therefore, DRG apoptosis is proposed as a primary mechanism of neurotoxicity induced by cisplatin, characterizing sensory neuronopathy [36,39]. However, the neurotoxicity may result in secondary axonal damage and chronic sensory neuropathy [36,40]. These neurotoxic effects can occur directly through interactions of chemotherapy drugs with all neuron structures or indirect mechanisms, such as inflammation and glial damage development [34]. Interestingly, postmortem tissue of cisplatin-treated patients demonstrated a reduced volume of DRG soma, evidence of necrosis, and large fibre axonal loss [41].

Clinically, the sensory symptoms of CIPN are predominant in patients and can persist for months to years after the completion of chemotherapy [38,42,43]. Generally, CIPN symptoms are more prominent in the distal extremities, such as the feet and hands, known as the “glove and stocking” distribution. However, prolonged treatment may worsen signs and symptoms and progress to more proximal areas of the limbs [10,27,42,44]. Patients manifest symptoms such as numbness and tingling (paraesthesia, an abnormal sensation that is not painful or unpleasant), which may occur spontaneously or when evoked by a stimulus. Additionally, characteristic symptoms of neuropathic pain are frequently reported, such as sensations like burning, tingling, freezing, and electric shock-like pain, as well as allodynia (pain to a stimulus that generally does not produce pain) or hyperalgesia (increased pain from a stimulus that usually induces pain) provoked by mechanical or thermal stimuli [10,39,42,44,45]. While sensory nerve dysfunction is prominent, in severe cases, the damage may extend to autonomic and motor nerves, thus inducing motor and autonomic signs and symptoms [27,29,42]. Consequently, this condition may require subtherapeutic dosing, dose interruptions or treatment discontinuation before completing the chemotherapeutic regimen. Understandably, this has implications for the effectiveness of oncological treatment and overall patient survival [42,46,47].

Although each chemotherapy drug has particularities in the development of CIPN, the predominant feature and risk factor among most classes is the dose-dependent nature of this condition. Specifically, the incidence of cisplatin-induced neuropathy highly depends on the cumulative dose, with mild to severe peripheral neurotoxicity being observed in adult patients after a cumulative dose of 300–350 mg/m^2^ [27,29,39]. Higher cumulative doses of cisplatin and longer exposure times increase the severity of CIPN, as well as the possibility of developing chronic neuropathy [10,39]. Furthermore, signs and symptoms may develop or worsen after treatment has stopped, a phenomenon known as “coasting” [29,39]. In particular, about 30–50% of individuals who completee the treatment course with cisplatin experience peripheral neuropathy, with 10% of them presenting disabling symptoms [37]. In addition to the dose of chemotherapy, the incidence of CIPN can be influenced by several other factors, such as pre-existing conditions, including diabetes, previous peripheral nerve damage, patient age, and the combination of neurotoxic chemotherapy agents, such as the combined therapy of cisplatin and paclitaxel [48].

Therefore, CIPN is one of the clinical complications of antitumour chemotherapy that seriously affects daily activities and impacts the overall quality of life of cancer patients and survivors [25,27,34]. Despite the severe and long-lasting symptoms associated with CIPN, there are currently no efficacious pharmacological and nonpharmacological interventions or preventive strategies to mitigate the development of peripheral neuropathy [43]. Even after more than two decades of research and several clinical trials, the only medication the American Society of Clinical Oncology recommends to treat CIPN is duloxetine, a serotonin-noradrenaline uptake inhibitor [43,47].

In recent years, a substantial portion of research has been dedicated to unravelling the mechanisms of CIPN and has primarily concentrated on the peripheral nervous system. These studies use experimental animals (rats or mice) submitted to chemotherapy over an extended period (days or weeks) to simulate the treatment process observed in humans. This approach is complemented by evaluations of clinical indicators of CIPN, including allodynia and hyperalgesia, and the assessment of possible mechanisms related to the development of CIPN. In this sense, numerous mechanisms are implicated in CIPN, such as changes in cell-signalling pathways such as G-coupled protein receptors (GPCRs), altered expression of ion channels, inflammatory response, and mitochondrial dysfunction.

Therefore, an updated literature review of the essential mechanisms underlying cisplatin-induced peripheral neuropathy is needed. This may be crucial for identifying new pharmacological approaches to help advance the management of CIPN symptoms without affecting the oncological treatment regimen. This review, therefore, concentrates on the role of GPCRs and ion channels in animal models of cisplatin-induced neuropathy. For this purpose, the review was carried out in the PubMed database. The search was performed in the PubMed database for indexed articles published in English by crossing the keyword cisplatin: “Cisplatin AND pain AND G protein-coupled receptors” and “Cisplatin AND pain AND ion channels”. The manuscripts were selected based on a careful review of the abstracts, centred only on studies using cisplatin as a single agent. Studies addressing other chemotherapies were not included unless the results were specifically related to cisplatin.

## 4. Role of G Protein-Coupled Receptors in Cisplatin-Induced Peripheral Neuropathy

GPCRs are involved in most physiological and pathophysiological processes, including pain. Substantially, GPCRs are widely expressed by sensory neurons, detecting both excitatory and inhibitory stimuli exerting actions on pain transmission [49,50].

GPCRs constitute the largest, most dynamic and characterized family of receptors, with approximately 850 members [51,52]. GPCRs are formed by seven transmembrane domains, an extracellular amino terminus, and an intracellular carboxyl terminus and can be activated by several ligands. To initiate signalling, GPCRs couple with intracellular transducers such as heterotrimeric G proteins, comprising Gα, Gβ, and Gγ subunits, and activating several downstream effector molecules. Gα subunits are classified into four subfamilies, Gαs, Gαi, Gαq, and Gα12/13, and each of these families activates unique signalling pathways. The stimulation of receptors coupled to Gαs activates adenylyl cyclase, stimulating their catalytic activity and thus cyclic adenosine monophosphate (cAMP) production and subsequent activation of cAMP-regulated kinase proteins such as protein kinase A (PKA) that sustains intracellular signals. Differently, the activation of Gαi proteins inhibit several adenylyl cyclase isoforms, reducing the intracellular cAMP levels. Members of the Gαq family interact with and activate the phospholipase C (PLC) pathway, with consequent formation of inositol 1,4,5- triphosphate (IP_3_) and diacylglycerol (DAG). IP_3_ stimulates the release of calcium (Ca^2+^) from intracellular stores, which, together with DAG, activates protein kinase C (PKC). The Gα12/13 family interact with members of the RH domain containing guanine nucleotide exchange factors for the Rho (RhoGEFs) family of proteins, leading to the subsequent activation of the Rho family of small GTPases [51,52].

**Figure 2:**
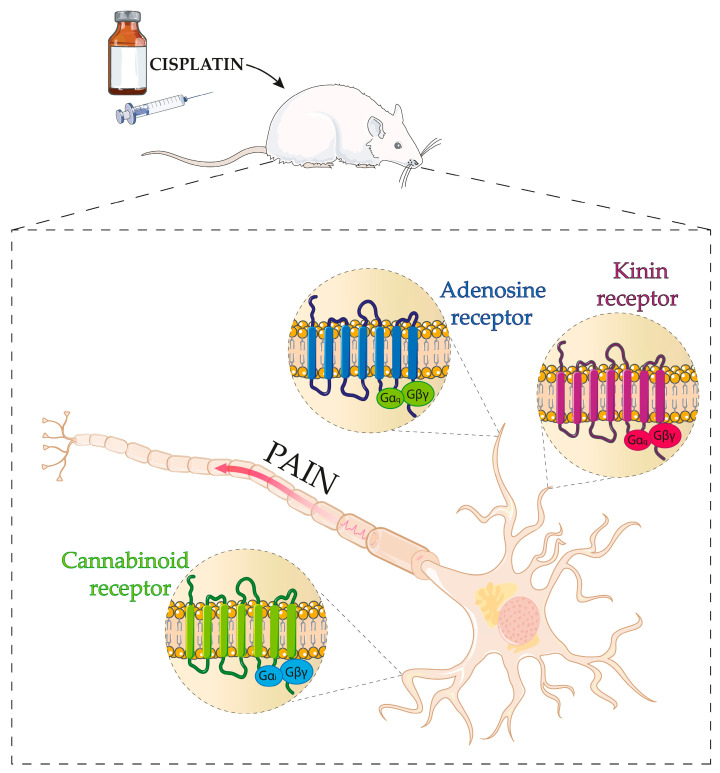
Schematic representation of the principal GPCRs involved in cisplatin-induced peripheral neuropathy. The Figure demonstrates kinin B_1_ and B_2_ receptors, adenosine receptors, and CB1 and CB2 receptors involved in cisplatin-induced peripheral neuropathy. Parts of the Figure were drawn using pictures from Servier Medical Art by Servier, licensed under a Creative Commons Attribution 3.0 unported license.

GPCRs and cascades downstream of their activation are implicated in cisplatin-induced peripheral neuropathy. Members of the GPCRs family, such as kinins receptors, are expressed in important structures for pain modulation in the peripheral and central nervous system [53]. In this sense, recent studies using pharmacological antagonism and genetic manipulation have demonstrated the contribution of kinin B_1_ and B_2_ receptors to the painful behaviours of cisplatin-induced peripheral neuropathy [54]. Furthermore, it was observed that PLC and protein kinase C epsilon (PKCε) intracellular signalling pathways downstream from the kinin B_2_ receptor activation are involved in the transient receptor potential ankyrin 1 (TRPA1) channel sensitization, contributing to the painful behaviours caused by cisplatin in mice [55]. Thus, kinin receptor antagonists present themselves as promising pharmacological strategies for treating cisplatin-induced peripheral neuropathy (Figure 2).

Adenosine is a purine nucleoside that signals via four different GPCRs (A_1_, A_2A_, A_2B_, and A_3_) in various tissues, especially in the DRGs, spinal cord, supraspinal structures, and immune cells [56,57]. They are, therefore, critical in physiological and pathophysiological states, including pain. In this sense, the modulation of adenosine receptors is essential for acute and chronic pain models [58]. Recently, Dewaeles et al., 2022, observed that the FDA-approved adenosine A_2A_ receptor antagonist, istradefylline (KW6002), attenuated cisplatin-induced painful hypersensitivity by reducing the expression of cytokines related to CIPN in DRGs. Favourably, KW6002 presented nephroprotective effects and did not alter the antitumour properties of cisplatin in tumour-bearing mice and could even enhance them. Given the safety of KW6002, the clinical translation would benefit cancer survivors and cancer patients undergoing cisplatin treatment [59]. In addition to the adenosine A_2A_ receptor, a study highlighted the role of the adenosine A_3_ receptor in regulating pain induced by cisplatin. Adenosine A_3_ receptor selective agonist, MRS5890, prevented signs of cisplatin-induced peripheral neuropathy, such as mechanical allodynia, spontaneous pain, and sensorimotor deficits, highlighting the efficacy of adenosine A_3_ receptor agonist in neuropathic pain induced by cisplatin [60] (Figure 2).

The endocannabinoid system is another system implicated in painful conditions that have received substantial attention in research. The modulation of this system has demonstrated promising outcomes in attenuating pain, such as CIPN, in several clinical and preclinical studies [61]. The endocannabinoid system is an essential physiological system, which encompasses its two receptors, cannabinoid receptor type 1 (CB1) and 2 (CB2), endogenous cannabinoid ligands, mainly anandamide (AEA) and 2-arachidonoyl-glycerol (2-AG), and metabolic enzymes responsible for metabolizing, such as fatty acid amide hydrolase (FAAH) and monoacylglycerol lipase (MGL). Both cannabinoid receptors, CB1 and CB2, belong to the GPCRs family and exert their actions primarily through the Gi protein. Regarding the expression of receptors, CB1 receptors are predominantly expressed throughout the central nervous system, while CB2 receptors are found primarily in immune cells and peripheral tissues [61,62]. In this sense, studies have demonstrated the importance of this system for pain in CIPN and specifically for pain caused by cisplatin (Figure 2).

Compounds targeting the endocannabinoid system have attenuated cisplatin-induced pain behaviours in rodents. Specifically, a selective agonist of CB1 receptors, ACEA, locally or systemically administered, decreased mechanical allodynia in cisplatin-treated rats [63]. Given the limitations of CB1 agonists related to psychoactive effects, a recently developed synthetic peripherally restricted cannabinoid, 4-{2-[-(1E)-1[(4-propylnaphthalen-1-yl)methylidene]-1H-inden-3-yl]ethyl}morpholine (PrNMI), has shown effectiveness in alleviating cisplatin-induced mechanical and cold allodynia without significant psychoactive adverse effects or tolerance to repetitive administration [64]. Furthermore, a CB1 receptor-positive allosteric modulator (GAT229) was also tested in cisplatin-induced peripheral neuropathy. GAT229 mitigated and slowed the progression of cisplatin-induced mechanical allodynia and heat thermal hyperalgesia without eliciting the typical manifestations associated with CB1 receptor activation, such as psychoactive effects, tolerance, and dependence [65]. Similarly, CB2 receptor agonists, such as AM1710 and JWH-133, have also been shown to suppress mechanical and cold allodynia in the cisplatin-induced neuropathic pain model [63,66].

Non-selective CB1/CB2 receptor agonists WIN55,212–2, delta-9-tetrahydrocannabinol, and CP55,940 also alleviated the signs of cisplatin-induced peripheral neuropathy [63,67,68,69]. Interestingly, these studies also show that tolerance to the antinociceptive effects of cannabinoid agonists can develop through desensitization of the CB1 following prolonged administration. Desensitization results from the phosphorylation of serine residues of the CB1 receptor by a G protein-coupled receptor kinase (GRK) and the subsequent receptor association with β-arrestin. Thus, mutation of these phosphorylation sites of CB1 leads to decreased desensitization and a prolonged antinociceptive effect of WIN55,212–2 in mice with cisplatin-induced chronic neuropathy. In contrast, this mutation did not delay the tolerance to delta-9-tetrahydrocannabinol and CP55,940 effects in cisplatin-induced neuropathy [63,67,68,69].

In addition to cannabinoid receptors, studies have demonstrated the importance of endocannabinoids AEA and 2-AG and the enzymes controlling degradation in cisplatin-induced peripheral neuropathy. The intraplantar administration of endocannabinoids AEA and 2-AG attenuated the cisplatin-established mechanical hyperalgesia in mice, acting through a CB1-dependent mechanism but not CB2 receptors [70,71]. Enzymes metabolize these endocannabinoids; FAAH hydrolyzes AEA, and MGL primarily hydrolyzes 2 AG. Consequently, the use of peripherally restricted (URB937) and centrally active (URB597) FAAH inhibitors reduced cisplatin-evoked behavioural hypersensitivities [70,72,73,74]. Likewise, MGL inhibitors, such as JZL184, also reduced the mechanical and cold allodynia induced by cisplatin [71,72]. Therefore, inhibitors of metabolizing enzymes present an effective pharmacological strategy for alleviating cisplatin-induced pain symptoms.

Repeated treatment with cisplatin causes changes in multiple endocannabinoid signalling elements in tissues related to transduction and transmission of nociception. Levels of CB2 mRNA were reduced in the DRG of rats administered cisplatin [64]. Correspondingly, CB2 protein expression was reduced by more than 75% in cisplatin-treated mice’s plantar paw skin and spinal cord [71]. In contrast, the expression of CB1 receptors was unaffected by mice cisplatin exposure [64,71]. Furthermore, treatment with cisplatin alters endocannabinoid tone; specifically, it was observed that cisplatin decreases 2-AG and AEA content in the DRG and plantar paw skin [70,71,72]. Conversely, it increases levels of both 2-AG and AEA in the lumbar spinal cord, potentially reflecting an adaptive central response to peripheral injury induced by chemotherapy [71,72]. Taken together, these data demonstrate the importance of the endocannabinoid system for peripheral neuropathy induced by cisplatin, showing how this system can be altered by cisplatin treatment, as well as being an alternative for the treatment of cisplatin-caused painful conditions. Table 1 provides an overview of the studies mentioned here.

## 5. Role of Ion Channels in Cisplatin-Induced Peripheral Neuropathy

Nociceptive neurons, responsible for detecting painful signals, express a wide array of ion channels, such as sodium, potassium, and calcium ion channels. Collectively, these channels regulate nociceptor excitability and function as pain transducers. In summary, voltage-gated sodium and potassium channels are crucial in generating action potentials, while voltage-gated calcium channels are essential for neurotransmitter release from central or peripheral nociceptor terminals [75,76].

**Figure 3:**
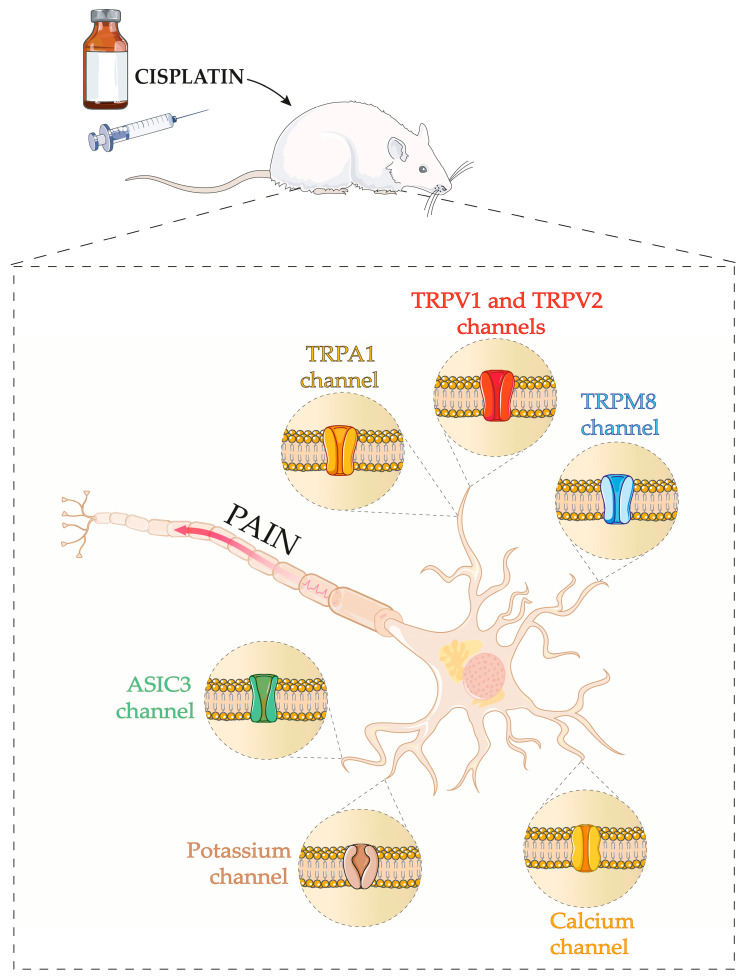
Schematic diagram of the principal ion channels involved in cisplatin-induced peripheral neuropathy. The Figure demonstrates TRPA1, TRPV1, TRPV2, TRPM8, ASIC3, potassium, and calcium channels involved in cisplatin-induced peripheral neuropathy. Parts of the Figure were drawn using pictures from Servier Medical Art by Servier, licensed under a Creative Commons Attribution 3.0 unported license.

In this sense, most of the time, the development of peripheral neuropathy associated with the use of chemotherapy drugs is related to changes in the activation potential of peripheral nerves, which may be linked to changes in the expression and function of ion channels, such as voltage-gated calcium channels, voltage-gated potassium channels, and transient receptor potential family (TRP) channels (Figure 3).

TRPs are channels widely expressed in sensory neurons and act as transducers for thermal, chemical, and mechanical stimuli, implicated in different painful conditions, including peripheral neuropathy induced by chemotherapy drugs [77]. In this sense, Ta et al. (2009) showed the first evidence regarding the contribution of the transient receptor potential vanilloid 1 (TRPV1) channel as an essential mediator for thermal hyperalgesia in cisplatin-induced neuropathy, in which cisplatin treated-TRPV1 knockout mice did not develop thermal hyperalgesia [78]. A recent study found similar results for the heat-evoked response in TRPV1 knockout mice, in which the development of thermal hyperalgesia induced by cisplatin was controlled for up to 2 weeks [79]. Beyond TRPV1, the TRPA1 channel contributes significantly to cisplatin-induced mechanical allodynia since TRPA1 null mice do not reduce mechanical threshold after cisplatin treatment [80].

In addition to genetic approaches, pharmacological tools suggest the involvement of TRPA1 and TRPV1 in cisplatin-induced pain behaviours. For example, the pharmacological inhibition of the TRPA1 channel (using the A967079 antagonist) reduced the mechanical and cold allodynia induced by cisplatin in mice, while a TRPA1 agonist (allyl isothiocyanate) at a subnociceptive dose enhanced these behaviours [55]. Furthermore, cisplatin-induced mechanical hypersensitivity was inhibited by silencing TRPA1 and TRPV1 through the intraplantar administration of a membrane-impermeable lidocaine analogue (QX-314) together with TRPV1 agonist (capsaicin) or with TRPA1 agonist, allyl isothiocyanate (AITC) [81].

In line with this finding, an up-regulation of TRPA1, TRPV1, and transient receptor potential melastatin 8 (TRPM8) mRNA was observed in DRG neurons cultured and treated with cisplatin [82]. A similar increase in TRPV1 and TRPA1 mRNA in DRG neurons occurred following in vivo treatment with cisplatin [82]. In agreement, Khasabova [70] observed increased TRPV1 expression after immunofluorescence in cisplatin-treated mice’s DRG neurons. This increase in the expression of TRP channels after cisplatin treatment may be correlated with cisplatin-induced mechanical and thermal hypersensitivity. On the other hand, there was no change in the proportion of TRPV1-immunopositive trigeminal ganglia neurons in cisplatin-treated mice [82]. Another study also did not identify changes in the TRPV1-positive cells in all DRG neurons or muscle-afferent neurons after cisplatin administration [83].Furthermore, this study showed no alteration in noxious thermal sensitivity, although TRPV2-positive cells significantly increased in cisplatin-treated rats [83].

Interestingly, the cytosolic calcium concentration increased in DRG neurons positive for TRPV1 and TRPA1 channels exposed to cisplatin, while the mitochondrial calcium concentration decreased, indicating a calcium release from mitochondria to the cytosol [84]. Besides regulating TRP channels, cisplatin also modulates voltage-gated calcium channels. This modulation changes the intracellular calcium homeostasis, a process critical for the induction of neurotoxicity.

The calcium channel family consists of several channel subtypes that can be divided broadly into two groups based on their voltage dependence of activation: low voltage-activated and high voltage-activated channels. The high voltage-activated calcium channels are heteromultimeric protein complexes comprised of a pore-forming α1 subunit and the auxiliary subunits, β, γ, and α2δ. The high voltage-activated calcium channels are further classified as voltage-gated calcium (CaV) of L-type (Ca_V_1.2, Ca_V_1.3, Ca_V_1.4), P/Q-type (Ca_V_2.1), N-type (Ca_V_2.2), and R-type (Ca_V_2.3). Three types of Ca_V_3 channels are low voltage-activated (Ca_V_3.1, Ca_V_3.2, and Ca_V_3.3), all of which represent T-type calcium channels [85]. Nociceptors also express different subtypes of potassium (K^+^) channels, which constitute the most diverse ion channel family, distinguished by their activation mechanisms and structures. They include several types of voltage-gated K^+^ channel (K_V_), calcium-activated K^+^ channel (KCa), sodium-activated K^+^ channel (KNa), two-pore domain K^+^ channel(K_2_P), and inwardly rectifying K^+^ channel (Kir) [76].

The first evidence showed that small DRG neurons exposed to cisplatin reduced the peak and sustained Ca^2+^ currents. These reductions in Ca^2+^ currents were through non-specific calcium channels; however, differential modulation of voltage-gated calcium channel current subtypes has been suggested [86]. In this sense, analogous research showed the essential role of N-type Ca_V_ channels in cisplatin-induced peripheral neuropathy for the first time. Firstly, this study identified a concentration-dependent effect of cisplatin on Ca_V_ channel currents, where acute exposure reduced Ca_V_ channel currents, while long-term exposure of DRG neurons to cisplatin increased Ca_V_ current densities, suggesting that acute and long-term effects of cisplatin may be mediated by specific subtypes of Ca_V_ channels [87]. Thus, specific measurements of Cav channel currents showed that the currents of P/Q- (Ca_V_2.1), L- (Ca_V_1.1–Ca_V_1.4), and T (Ca_V_3.1–Ca_V_3.3)-type Ca_V_ channels were reduced, and those of N-type (Ca_V_2.2) Ca_V_ channels were increased under cisplatin treatment, endorsing the idea that the N-type Ca_V_ channel may be related to increased intracellular calcium during cisplatin treatment [87]. Interestingly, there are differences in how N-type currents were upregulated. Short-term cisplatin exposure increased N-type Ca_V_ currents via PKC activation. In contrast, the calcium/calmodulin kinase II activation mediated the N-type Ca_V_ current density increases after long-term cisplatin exposure [87]. Therefore, channel regulation can occur through a short-term mechanism via the phosphorylation of the ion channel by PKC. Conversely, long-term regulation may be overexpression of the N-type Ca_V_ channel mediated by calcium/calmodulin kinase II.

Beyond the functional modulation, cisplatin increased the N-type Ca_V_ channel protein expression in vitro and in vivo without influencing the mRNA level in DRG neurons. Similarly, repetitive in vivo cisplatin administration (three injections) increased N-type protein expression for at least 14 days after the last cisplatin administration [88]. Since Ca_V_ channels are essential for calcium influx into neuronal cells and the upregulation of these channels may contribute to a critical increase of intracellular calcium, this observed protein overexpression may be responsible for increasing N-type Ca_V_ channel current density [87,88]. Moreover, auxiliary subunits important for Ca_V_ channel function and expression, specifically α1β and α2δ1 subunits of the N-type Ca_V_ channel, were also increased in DRG after cisplatin exposure [87]. In accordance with this data, gabapentin and pregabalin, drugs that bind and inhibit the α2δ1 subunit of Ca_V_ channels, have been shown to diminish neuropathic-like pain associated with cisplatin [89,90,91,92,93]. These data reinforce that the Ca_V_ channels contribute to cisplatin-induced peripheral neuropathy, especially the Ca_V_1 and Ca_V_2 channel subtypes.

Further evidence supports the role of N-type Ca_V_ channels in symptoms of cisplatin-related neuropathic pain. Using an N-type Ca_V_ blocker, ω-conotoxin MVIIA, administered intravenously, prevented typical signs of cisplatin-induced peripheral neuropathy as heat hyperalgesia and mechanical allodynia [87,88]. Notably, ω-conotoxin was approved by the Food and Drug Administration for human use and is clinically available as ziconotide (Prialt^®^), administered via intrathecal infusion to treat severe chronic pain [94]. Furthermore, ω-conotoxin MVIIA prevented the cisplatin-mediated increase in N-type protein expression without a present effect on N-type channel expression and behavioural characteristics when ω-conotoxin MVIIA was administrated alone [87]. Therefore, the N-type Ca_V_2.2 channel blockade presents direct neuroprotective effects over the behavioural characteristics and N-type channel expression induced by cisplatin, thus representing a new neuroprotective strategy against the neurotoxic actions of cisplatin. Despite the well-established efficacy of ω-conotoxin MVIIA administered via the intrathecal route in the cisplatin-induced neuropathy model, recent observations indicate that subcutaneous administration (intraplantar) of three distinct ω-conotoxins—MVIIA, GVIA, and CVIF—failed to reduce mechanical allodynia in a model of acute peripheral neuropathy induced by intraplantar cisplatin administration in mice [95].

In addition to voltage-gated calcium channels, evidence supports a possible role of voltage-gated potassium channels in chemotherapy drugs-evoked neuronal hyperexcitability. In this sense, it was observed that a Kv7 channel activator, retigabine, partially prevented the membrane depolarization and peripheral axon loss in cisplatin-treated mice. Thus, it is assumed that the retigabine effect in causing an increase in K^+^ currents produce a hyperpolarizing shift in membrane potential, which may partially contribute to the reduction of cisplatin-induced neuropathic pain [96].

Recently, a reduction of Kir4.1 expression and current density was shown in cultured satellite glial cells exposed to cisplatin. Since Kir4.1 is an essential protein in regulating neuronal activity, the reduced Kir4.1 expression and its functional modulation after cisplatin exposure suggest a critical role for this channel in peripheral neuropathy [97]. Furthermore, cisplatin reduced the current density in K2P18.1- and Kv1.4-transfected HEK 293 cells. Thus, K2P18.1, a two-pore K^+^ channel, is another K^+^ channel through which cisplatin may confer neuronal hyperexcitability [98]. Therefore, the regulation of these channels could be a promising alternative to reduce cisplatin-induced neuropathic pain.

The participation of acid-sensitive ion channels (ASICs), particularly ASIC3, has been noted in the model of cisplatin-induced peripheral neuropathy. Hori et al. showed that ASIC3 plays an essential role in mediating mechanonociception. Cisplatin administration increased ASIC3 expression in small and medium-sized cells of all DRG neurons, as well as in a group of medium-sized cells of L5 DRG neurons innervating the gastrocnemius muscle. Furthermore, using an ASIC antagonist, amiloride, suppressed cutaneous and muscular hyperalgesia induced by cisplatin administration [83]. Hence, this study demonstrates the implication of ASIC3 in developing cisplatin-induced mechanical hypersensitivity. Table 2 provides an overview of the studies mentioned here.

In summary, the employment of animal models for peripheral neuropathy induced by cisplatin has revealed that cisplatin treatment can influence the function and change the expression of various ion channels. Among these are TRP channels, voltage-gated calcium and potassium channels, and ASICs, which are implicated in structures crucial for nociceptive processing. Therefore, numerous studies have demonstrated the impact of various molecules, whether derived from natural sources or not, with activity on these channels in the search for novel therapeutic interventions for treating cisplatin-induced peripheral neuropathy, particularly pain. In this context, as previously described, the investigations involving ω-conotoxins are an example. Another example is the natural compound Berberine, which prevented and reduced painful behaviours induced by the peripheral neuropathy model with cisplatin. The observed effects of berberine are suggested to be linked to indirect effects on TRPV1 activity [79]. Similarly, the flavonoid 6-Methoxyflavanon has been shown to mitigate cisplatin-induced nociceptive behaviours. These effects are related to the influence of 6-Methoxyflavanon on GABAergic receptors [99,100]. 

## 6. Conclusions

Neurotoxicity is a substantial dose-limiting factor in cisplatin treatment. Animal studies have delineated many molecular mechanisms related to the peripheral neuropathy induced by cisplatin. CIPN is multifactorial, with impacts on DNA, neuroimmune mechanisms, ion channel expression and function, and changes in GPCR activity. In this review, we highlight the involvement of ion channels and GPCRs in cisplatin-induced peripheral neuropathy and demonstrate how these may represent potential targets for studying new therapeutic agents for this condition to provide safe and effective treatments to overcome the effects of neurotoxicity, enabling the complete treatment of cancer and improving the patient’s quality of life during treatment and after oncological treatment.

## Figures and Tables

**Table 1:** In vivo preclinical studies of GPCRs involvement in cisplatin-induced peripheral neuropathy model.

Targets	Intracellular G Protein Subtypes	Pharmacological and Genetic Approaches	Observed Effects	References
Kinin B_1_ receptor	Gq	DALBk	Reduced mechanical and cold allodynia	Becker et al. [54,55]
		DABk	Enhanced mechanical allodynia	Becker et al. [54]
		Antisense oligonucleotides	Attenuated mechanical allodynia	Becker et al. [54]
Kinin B_2_ receptor	Gq	Icatibant	Reduced mechanical and cold allodynia	Becker et al. [54,55]
		Bk	Enhanced mechanical allodynia	Becker et al. [54]
		Antisense oligonucleotides	Attenuated mechanical allodynia	Becker et al. [54]
Adenosine _2A_ receptor	Gq	Istradefylline (KW6002)	Reduced mechanical hypersensitivity	Dewaeles et al. [59]
Adenosine _3A_ receptor	Gq	MRS5890	Prevented cisplatin-induced mechanical allodynia and spontaneous pain	Singh et al. [60]
Cannabinoid receptor type 1 (CB1R)	Gi	ACEA	Alleviated mechanical allodynia	Vera et al. [63]
		4-{2-[-(1E)-1[(4-propylnaphthalen-1-yl)methylidene]-1H-inden-3-yl]ethyl}morpholine (PrNMI)	Attenuated mechanical and cold allodynia	Mulpuri et al. [64]
		GAT229	Attenuated and slowed progression of mechanical allodynia and heat hyperalgesia	Bagher et al. [65]
Cannabinoid receptor type 2 (CB2R)	Gi	AM1710	Attenuated mechanical and cold allodynia	Deng et al. [66]
		JWH-133	Reduced mechanical allodynia	Vera et al. [63]
			Reduced mRNA levels of CB2R in the DRG	Mulpuri et al. [64]
			Reduced CB2R protein in the skin paw and spinal cord	Khasabova et al. [71]
Cannabinoid receptor type 1 and 2	Gi	URB597, URB937 and JZL184	Reduced mechanical and cold allodynia	Guindon et al. [72] Khasabova et al. [71] Khasabova et al. [70] Thompson et al. [73] Uhelski et al. [74]
		WIN55,212–2	Attenuated mechanical allodynia	Nealon et al. [67]
		Delta-9-tetrahydrocannabinol	Reduced mechanical allodynia	Henderson-Redmond et al. [69]

Abbreviations—ACEA: selective CB1R agonist; AM1710: CB2R agonist; Bk: bradykinin (kinin B_2_ receptor agonist); cannabinoid type 1 receptor (CB1R); cannabinoid type 2 receptor (CB2R); DABk: des-Arg^9^-bradykinin (kinin B_1_ receptor agonist); DALBk: des-Arg^9^[Leu^8^]-bradykinin (kinin B_1_ receptor antagonist); DRG: (Dorsal Root Ganglia); GAT229: CB1R-positive allosteric modulator; JZL184: monoacylglycerol lipase (MGL) inhibitor; JWH-133: CB2R agonist; URB937: peripheral fatty acid amide hydrolase (FAAH) inhibitor; URB597: central FAAH inhibitor; WIN55,212–2: non-selective CB1/CB2 receptor agonist.

**Table 2:** In vivo preclinical studies of ion channels involvement in cisplatin-induced peripheral neuropathy model.

Targets	Pharmacological and Genetic Approaches	Observed Effects	References
TRPV1 channel	C57Bl6 *TRPV1*^−/−^	Prevented the development of heat hyperalgesia	Ta et al. [80]; Meng et al. [81]
	QX-314	Inhibition of mechanical hypersensitivity	Shim et al. [83]
		Increased the mRNA levels in DRG neuron culture	Ta et al. [80]
		No change in the TRPV1 immunoreactivity profiles in DRG and trigeminal ganglia neuron	Khasabova et al. [70] Ta et al. [80]
		No changes in TRPV1-positive cells of DRG and trigeminal ganglia neurons	Hori et al. [84]
TRPA1 channel	C57Bl6 *TRPA1*^−/−^	Avoided the development of mechanical allodynia	Nassini et al. [82]
	Allyl isothiocyanate	Increased mechanical allodynia	Becker et al. [55]
	A967079	Attenuated mechanical allodynia	Becker et al. [54]
	QX-314	Inhibition of mechanical hypersensitivity	Shim et al. [83]
		Increased TRPA1 mRNA levels in DRG neuron culture	Ta et al. [80]
TRPM8 channel		Increased mRNA levels in DRG neurons culture	Ta et al. [80]
TRPV2 channel		Increased TRPV2 expression in DRG neurons	Hori et al. [84]
N-type Ca_V_ channels	ω-conotoxin MVIIA	Increased channel protein expression in DRG neurons	Leo et al. [88]
		Increased the expression of α1β and α2δ1 subunits in DRG neurons	Leo et al. [87]
		Prevented the development of mechanical allodynia and heat hyperalgesia	Leo et al. [87,88]
	ω-conotoxins MVIIA, GVIA, and CVIF	Failed to reduce mechanical allodynia after intraplantar administration	Hasan et al. [95]
K_V_7 channel	Retigabine	Prevented membrane depolarization and peripheral axon loss	Nodera et al. [96]
ASIC3		Increased ASIC3 protein expression	Hori et al. [84]
	Amiloride	Alleviated cutaneous and muscular hyperalgesia	Hori et al. [84]

Abbreviations—ASIC: acid-sensitive ion channel; A967079: TRPA1 channel antagonist; DRG: (Dorsal Root Ganglia); QX-314: lidocaine analogue; TRPA1: transient receptor potential ankyrin 1; *TRPA1*^−/−^: *TRPA1* knockout mice; TRPM8: transient receptor potential melastin 8; TRPV1: transient receptor potential vanilloid 1; *TRPV1*^−/−^: TRPV1 knockout mice; TRPV2: transient receptor potential vanilloid 2.

## Abbreviations

HIV—human immunodeficiency virus; CIPN—chemotherapy-induced peripheral neuropathy; IASP—International Association for the Study of Pain; ICD-11—International Classification of Diseases; DRG—dorsal root ganglia; GPCR—G-coupled protein receptors; cAMP—cyclic adenosine monophosphate; PKA—protein kinase A; PLC—Phospholipase C; PKC—protein kinase C; IP3—Inositol triphosphate-3; DAG—Diacylglycerol; RhoGEFs—Guanine nucleotide exchange factors for the Rho; PKCε—protein kinase C epsilon; TRP—transient receptor potential; TRPA1—transient receptor potential ankyrin 1; TRPV1—transient receptor potential vanilloid 1; TRPV2—transient receptor potential vanilloid 2; TRPM8 –transient receptor potential melastin 8; AEA—Anandamide; CB1—cannabinoid receptor type 1; CB2—cannabinoid receptor type 2; 2-AG—2-arachidonoyl-glycerol; MGL—Monoacylglycerol lipase; FAAH—fatty acid amide hydrolase; GRK—G protein-coupled receptor kinase; K^+^—potassium; KCA—calcium-activated K^+^ channels; K2P—two-/tandem-pore domain; Kir—inwardly rectifying; CaV—voltage-gated calcium channel; Kv—voltage-gated K^+^ channel; Ca^2+^—calcium; ASIC—acid-sensitive ion channels.
